# Mesenchymal Stem Cells in Aplastic Anemia and Myelodysplastic Syndromes: The “Seed and Soil” Crosstalk

**DOI:** 10.3390/ijms21155438

**Published:** 2020-07-30

**Authors:** Bruno Fattizzo, Juri A. Giannotta, Wilma Barcellini

**Affiliations:** 1 Hematology Unit, Fondazione IRCCS Ca’ Granda Ospedale Maggiore Policlinico, 20100 Milan, Italy; juri.giannotta@unimi.it (J.A.G.); wilma.barcellini@policlinico.mi.it (W.B.); 2 Department of Hematology and Onco-Hematology, Università degli Studi, 20100 Milan, Italy

**Keywords:** aplastic anemia, myelodysplastic syndromes, mesenchymal stem cells, bone marrow, microenvironment, hematopoietic stem cell transplant

## Abstract

There is growing interest in the contribution of the marrow niche to the pathogenesis of bone marrow failure syndromes, i.e., aplastic anemia (AA) and myelodysplastic syndromes (MDSs). In particular, mesenchymal stem cells (MSCs) are multipotent cells that contribute to the organization and function of the hematopoietic niche through their repopulating and supporting abilities, as well as immunomodulatory properties. The latter are of great interest in MDSs and, particularly, AA, where an immune attack against hematopoietic stem cells is the key pathogenic player. We, therefore, conducted Medline research, including all available evidence from the last 10 years concerning the role of MSCs in these two diseases. The data presented show that MSCs display morphologic, functional, and genetic alterations in AA and MDSs and contribute to immune imbalance, ineffective hematopoiesis, and leukemic evolution. Importantly, adoptive MSC infusion from healthy donors can be exploited to heal the “sick” niche, with even better outcomes if cotransplanted with allogeneic hematopoietic stem cells. Finally, future studies on MSCs and the whole microenvironment will further elucidate AA and MDS pathogenesis and possibly improve treatment.

## 1. Introduction

Mesenchymal stem/stromal cells (MSCs) are multipotent cells that may be isolated from the bone marrow, umbilical cord blood, placenta, or adipose tissue [1,2] and they contribute to the organization and functioning of the hematopoietic niche [3,4,5]. According to the International Society for Cellular Therapy, the term “mesenchymal” is used for cells that are plastic-adherent in culture and express CD73, CD90, and CD105, but not CD14, CD34, CD45, CD79α, and human leukocyte antigen-D-related (HLA-DR). MSCs are able to differentiate into fibroblasts, osteoblasts, adipocytes, and chondroblasts [6] and to transdifferentiate to tissues of neuroectodermal origin (i.e., neurons or glial cells). In bone marrow (BM), endothelial cells are a known source of MSCs [7] and regulate hematopoietic stem cell (HSC) proliferation and differentiation by tight spatial colocalization with perivascular cells [8] and through secretion of E-selectin [9], cytochemokines, [10], and crosstalk molecule expressions such as Jagged1 and CXCL12 [11,12,13]. In the niche, MSCs also interact with the adrenergic fibers of the autonomic nervous system to regulate hematopoiesis, as described in several hematologic diseases [14,15].

**Figure 1:**
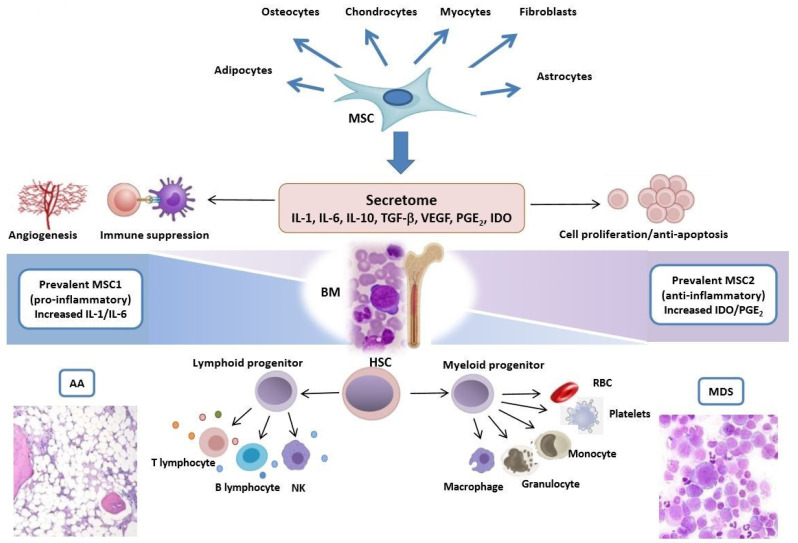
Multiple functions of mesenchymal stem cells in aplastic anemia and myelodysplastic syndromes. Mesenchymal stem/stromal cells (MSCs) are multipotent cells that are able to differentiate and transdifferentiate in different tissues (e.g., bone, cartilages, adipose tissue). They exert a supportive and modulating effect on the bone marrow (BM) niche and on innate and adaptive immunity via the secretion of soluble mediators (secretome). Various MSC alterations are described in AA and MDSs, including increased immunosuppression, decreased angiogenesis (prevalent in the former) and deregulated proliferation/apoptosis (prevalent in the latter). These phenomena may be mirrored by a phenotypic shift from type 1 (proinflammatory/immunosuppressive) to type 2 (anti-inflammatory/tumor-educated) MSCs along the clinical spectrum of BM failure syndromes. IL-1, interleukin-1; IL-6, interleukin-6; IL-10, interleukin-10; TGF−β, transforming growth factor-β; PGE_2_, prostaglandin E_2_; IDO, indoleamine 2,3-dioxygenase; MSC 1/2, type 1/2 MSC; HSC, hematopoietic stem cell; NK, natural killer; RBC, red blood cell.

Several studies have shown that MSCs have systemic immunoregulatory and immunosuppressive properties [16,17,18,19,20]. For instance, MSCs express cell surface molecules with an immunosuppressive capacity, such as programmed death ligand 1 (PD-L1) and Fas ligand [21,22]. Moreover, MSCs interact with T- and B-cells, NK cells, monocyte-derived dendritic cells, and neutrophils by direct cell-to-cell adhesion and/or secretion of soluble molecules, the so-called MSCs’ secretome. Among the various mediators, it is worth mentioning the key molecules involved in both BM niche physiology and pathology, such as interferon-γ (IFN-γ), interleukin-1β (IL-1β), transforming growth factor (TGF)-β1, indoleamine-2,3-dioxygenase (IDO), IL-6, IL-10, prostaglandin-E2, hepatocyte growth factor, tumor necrosis factor (TNF)-α, nitric oxide (NO), heme oxygenase-1, and HLA-G5. Concerning innate immunity, MSCs display anti-inflammatory properties by suppressing neutrophil respiratory bursts and prolonging granulocyte survival through IL-6 and STAT-3 signaling. Moreover, they inhibit mature dendritic cell differentiation by decreasing the expression of toll-like receptors 3 and 9 [23]. Finally, MSCs suppress NK proliferation and cytotoxicity. Regarding adaptive immunity, MSCs may repress T-helper type 1 (Th1) and Th17 polarization [24,25,26]. Specifically, they are able to induce anergy of activated T-cells and to elicit T-regulatory cell (Tregs) expansion via IL-10 secretion. Finally, B-cell proliferation/differentiation is also suppressed both directly and through activated CD4+ T-cell suppression [1] (Figure 1).

Several alterations of MSCs have been described in bone marrow failures, namely, aplastic anemia (AA) and myelodysplastic syndromes (MDSs), and it is not clear whether they are the cause or consequence of disease development and progression. Furthermore, niche disruption might sustain pancytopenia and promote the accumulation of clonal molecular alterations that lead to leukemic evolution. In addition, microenvironment alterations might, in turn, be potential targets for novel biologic drugs [1,27]. In this review, we will analyze the role of the microenvironment in AA and MDS pathogenesis and therapy, focusing on MSCs and their alterations. All studies dealing with mesenchymal cells will be discussed, including those papers where their stem cell features were not evidenced (mesenchymal stromal cells).

## 2. The Role of Mesenchymal Stem Cells and Microenvironment in Aplastic Anemia

AA is a rare hematologic disease caused by an immune attack to HSCs, either triggered by infectious agents (i.e., hepatitis viruses, HIV) or idiopathic T-cell-mediated marrow destruction as the leading pathogenic mechanism. BM lymphocytes from AA patients are able to inhibit HSCs, and oligoclonal expansion of dysregulated CD8+ T-cells has been demonstrated. Moreover, Th17 overexpression and concomitant Treg suppression [28,29,30,31,32], along with increased levels of proinflammatory cytokines such as IFN-γ and TNF-α at the molecular level, have been reported to play a key pathogenic role [33,34].

The reported incidence is 0.6–6.1 cases per million. These cases are diagnosed with a bone marrow biopsy showing various degrees of hypocellularity, with no signs of myelodysplasia, leukemia, and myelofibrosis, and the diagnosis requires the exclusion of secondary causes. Clinically, it is marked by peripheral cytopenias, with consequent fatigue, bleeding, and infections [35]. Disease severity is assessed by Camitta criteria that identify severe AA based on marrow cellularity <25% and the degree of cytopenias (PLT < 20 × 10^9^/L, ANCs < 0.5 × 10^9^/L, reticulocytes < 1%) or very severe AA if neutrophils are less than 0.2 × 10^9^/L. During workup, paroxysmal nocturnal hemoglobinuria (PNH) may be detected by flow cytometry, and reduced telomere length has been recently reported, particularly in cases aged ≥ 45 years [35]. Prompt intervention is usually required, and, along with close and careful transfusion support, a hematopoietic stem cell transplant (HSCT) from an HLA-matched sibling donor is the first choice for young (<40 years) patients. If a donor is unavailable or the patient is aged >40 years, the combination of rabbit/horse-derived antilymphocyte globulin (ATG) and ciclosporin (CyA) is the gold standard first-line immunosuppressive therapy [35]. About 1/3 of cases will require a second-line, including HSCT, a second course of immunosuppressive therapy, oral androgens, or the thrombopoietin analog eltrombopag. Survival is mainly age-related and also depends on the response to therapy [28,35].

### 2.1. Pathogenic Mechanisms of Mesenchymal Stem Cells in AA

The immunopathological mechanisms of aplastic anemia have shown to be modulated by MSCs and their secretome. Consistently, defects in the BM niche, including adipocytes, vascular cells, and MSCs have been demonstrated in AA. As shown in Table 1, the functioning, immunomodulating abilities and genetic features of AA–MSCs differ from healthy controls [27]. Regarding the contribution of MSCs to angiogenesis, Lu et al. showed a defective expression of vascular cell adhesion molecule-1 (CD106) in AA versus healthy donor MSCs. This resulted in a reduced and delayed capability of endothelial differentiation and BM vascularisation. Specifically, the marrow content of CD105 + CD106 + MSCs was significantly decreased, and the capability of CD31 + cell differentiation and tubular structure formation was delayed [36]. A more recent study on BM-derived MSCs demonstrated a significantly inhibited vascular endothelial growth factor (VEGF)–Notch signaling, with reduced proliferation and increased apoptosis. The latter could be reverted by VEGF–Notch axis stimulation in vitro [37]. The osteogenic potential of AA–MSCs was found to be reduced, whilst the adipogenic potential was increased. Recently, Li et al. identified novel microRNAs (miRNAs) that regulate AA–MSC differentiation [38]. Particularly, miR-144-3p was upregulated in BM-derived MSCs from AA patients, and its deletion enhanced osteogenic differentiation, whilst its overexpression halted the process. Interestingly, miR-144-3p is a negative regulator of ten-eleven translocation 2 (TET2), whose silencing was experimentally able to inhibit osteogenic differentiation. This may be a potential therapeutic target, by both enhancing TET2 signaling or inhibiting miR-144-3p.

MSCs’ immunomodulating properties are also implied in AA pathogenesis. BM-derived MSCs, intravenously injected into 15 patients, were shown to regulate Treg/Th17 balance [39], as well as their related cytokines. This happened through the remodulation of the Notch/RBP-J/FOXP3/RORγt pathway. Moreover, in a murine model, Zhao et al. demonstrated that human gingiva tissue-derived MSCs (GMSCs), may reinduce a balance between Th1/Th17 and Tregs by decreasing the former and increasing the latter, improving survival and attenuating AA phenotypes [40].

Regarding genetic studies, transcriptome analyses have demonstrated altered expressions of genes involved in cell proliferation, division, cycling, chemotaxis, interactions with HSCs, adipogenesis, and immune response in AA patients versus healthy controls [41]. In particular, Huo et al. systematically evaluated BM-derived MSCs from 39 healthy donors (HD) and 64 AA patients (including 15 BM). They found that AA–MSC morphology was different from HD–MSCs (large and swollen compared with a spindle shape), whilst immunophenotype was comparable. Quantitative RT–PCR analysis showed increased adipogenic potential (higher levels of *ADIPOQ* and *PPAR-γ*) and decreased osteogenic potential (decreased *RUNX2* and *BGLAP*). Furthermore, genomewide RNA sequencing showed a total number of 19,138 differentially expressed genes, and those upregulated in AA were involved in immune response (i.e., TNF, toll-like receptor, IL-17), cell division, cell adhesion (i.e., cell adhesion molecules (CAM), cell cycle, and differentiation. Finally, spliceosome analysis showed different spliceosomes with regard to histone deacetylase activity, cell growth, and various pathways (i.e., Wnt, mTOR, Hippo, Notch, and VEGF) in AA–MSCs versus HD–MSCs. Contrarily, single nucleotide polymorphisms and insertion–deletion were comparable between the two groups [42]. Although not conclusive, this very comprehensive study provides a basis for further investigations.

Finally, Bueno et al. found no differences between AA–BM–MSCs and those from healthy controls with regard to their support of hematopoiesis, immunosuppressive and anti-inflammatory properties [43], in contrast to the abovementioned studies. These discrepancies might be attributed to the heterogeneity of patients, particularly in old studies that may have also included misrecognized congenital syndromes such as Fanconi anemia and the Shwachman–Diamond syndrome. In the latter, MSCs and HSCs carry the same genetic lesion and may be hypothesized to both contribute to bone marrow failure. Two recent studies on MSCs demonstrated chromosomal fragility and early senescence in Fanconi anemia [44,45] and decreased angiogenic ability in the Shwachman–Diamond syndrome [46]. MSCs’ alterations further induce oxidative stress and DNA damage responses, promoting leukemogenesis in these diseases [47]. These are interesting models to speculate on the different contributions of MSC and HSC alterations in niche malfunctioning, leading to bone marrow failure. It has been speculated that MSC impairment in acquired AA may be predominantly “quantitative” (i.e., reduced number and function) as compared to the “qualitative” (i.e., genetic lesions/clonality) impairment observed in Fanconi anemia or SDS, suggesting that the latter may contribute to the higher leukemic tendency observed in these congenital syndromes [27].

### 2.2. Therapeutic Employment of MSCs in AA

MSCs may exhibit a therapeutic effect in hematologic diseases through transdifferentiation, immunomodulatory activity, and autocrine and paracrine effects on HSCs [48,49].

The strongest evidence is in regard to their use in the context of HSCT to facilitate engraftment, treat graft-versus-host disease (GVHD), and stimulate tissue repair [50]. Recently, De Lima et al. studied the effect of cotransplantation of allogeneic MSCs and unmanipulated cord blood units containing cells with hematopoietic activity in 31 adults with hematologic cancers and compared the results with 80 controls receiving cord blood HSCs only. They demonstrated that both neutrophil and platelet engraftments were more rapid and more likable (88% vs. 53% for neutrophils and 71% vs. 31% for platelets) in patients receiving MSCs [51]. This is in line with the demonstration of persistent aberrant MSCs in recipients after HSCT in AA, which are unable to repopulate the niche [52]. Specifically, to treat AA, both the elimination of auto-reactive T-cells and the regeneration of the progenitors are required. These goals may be supported by normal MSC infusions, given their immunosuppressive and regenerative properties. In the next paragraphs, the available studies on the therapeutic role of healthy MSCs in AA will be discussed (Table 2).

#### 2.2.1. MSC Infusions in AA Patients

Three studies have reported the efficacy and safety of BM-derived MSC infusions in relapsed/refractory AA patients. Two of the studies included a small number of patients, and concomitant immunosuppression was allowed; response rates were 20–30%, mainly partial, although with a favorable safety profile [53,54]. The third study evaluated 74 patients and documented a response in about 1/3 of them, with excellent overall survival at 17 months. Interestingly, previous use of ATG and the absence of infections emerged as predictors for response [55]. Altogether, these data suggest that MSCs could create a more favorable marrow microenvironment, permitting the recovery of hematopoietic progenitors in AA.

#### 2.2.2. MSC and HSC Cotransplantation in AA Patients

On the whole, we found 11 studies on MSC and HSC cotransplantation in relapsed/refractory AA, performed in the last 10 years. Five studies evaluated the cotransplantation of haploidentical BM HSCs and umbilical cord blood/BM-derived MSCs. All studies showed the reconstitution of platelets and granulocytes within 15 days, an incidence of grade III/IV acute GVHD of 12% to 29%, and severe chronic GVHD in less than 15% of the cases. Overall survival (OS) ranged from 75% to 84%, with some studies reaching a 5-year follow up [56,57,58,59,60]. In 2019, Zhao et al. [61] studied the combination of peripheral blood HSCs from unrelated (15) and related donors (10) and MSC infusion: most patients were engrafted and OS at 5 years was >80%. Fairly better results were reported in children receiving haploidentical or matched unrelated donor HSCs (both blood- and marrow-derived) together with MSCs. Very few grade III–IV GVHDs occurred, and excellent OS was documented [62,63,64]. Finally, Yue C. and colleagues [65] explored cotransplantation as the first-line in 6 patients: all achieved sustained, full donor chimerism, and only one patient died after a median follow-up of 21 months.

Regarding the risks of using MSCs, all studies showed that they are well-tolerated and safe for patients. No immediate infusional or late MSC-associated toxicities were observed in clinical trials on children and adults. The risk of infectious episodes related to MSC-induced immunosuppression is difficult to dissect in the context of HSCT. Furthermore, the follow-up of the various studies might be inadequate to evaluate the risk for MSC neoplastic transformation. However, most reports included a control group, and major infectious events, secondary neoplasms, or malignancy relapses did not seem to increase after MSC infusions, as also shown in a recent meta-analysis [66]. On the whole, treatment with MSCs seems promising, but results from clinical studies are still equivocal: about ¼ of cases will still suffer from severe acute or chronic GVHD, and more than 1/5 will die before the follow-up. To predict response, Hinden et al. evaluated sequential blood samples of 26 transplanted patients treated with MSCs. They found that a higher level of lymphocytes, particularly T- and NK-cells, may predict a good response to cotransplantation. The response also correlated with low levels of IL-6 and IL-22, Th17-related cytokines, prior to therapy [67].

#### 2.2.3. Other Studies on Therapeutic Use of MSCs in AA

As stated above, AA–MSCs have higher adipogenic potential. Liu et al. showed that levamisole is able to increase the expression of ZFP36L1, which functions as a negative regulator of MSC adipogenic differentiation [68]. Similar effects were obtained by using cyclosporin A, whose role on MSCs is so far unknown. Cyclosporin was able to suppress adipogenic differentiation of murine MSCs, inhibit IL-6 expression, and to promote programmed death-ligand 2 expression [69].

## 3. The Role of Mesenchymal Stem Cells in Myelodysplastic Syndromes

Myelodysplastic syndromes are a heterogeneous group of clonal disorders affecting HSCs, characterized by ineffective hematopoiesis with bone marrow dysplasia and various degrees of peripheral cytopenias [70]. A role for the immune system is reckoned, particularly in patients with hypocellular bone marrow. In these cases, autoimmune phenomena have been described, which may worsen the degree of cytopenia (particularly, anemia and thrombocytopenia) and respond to immunosuppressive therapy (i.e., steroids, cyclosporin) [71,72]. MDSs bear an intrinsic risk of evolution to acute leukemia, which on the whole, is estimated to be about 30% but differs according to the International Prognostic Scoring System (IPSS) and the presence of specific somatic mutations [73,74]. MDSs are typically a disease of an aged population, having an approximate incidence of 3–4/100,000/year, which rises to around 30/100,000/year among patients older than 70 [75]. The diagnosis is based on the presence of persistent cytopenia (hemoglobin < 100 g/L, absolute neutrophil count < 1.8 ×10^9^/L, platelet count < 100 × 10^9^/L), > 10% dysplasia in any hematopoietic lineage, and blast excess or MDS-defining cytogenetic abnormalities (reported in about 50% of patients). The outcome is extremely variable, with median survival ranging from over 5 years to less than 6 months, according to the prognostic scores [76]. Current treatment options are different for low- and high-risk MDSs, including erythropoiesis-stimulating agents, danazol and lenalidomide, for the former [77] and hypomethylating agents or HSCT for the latter [78]. New drugs aiming at targeting the aberrant HSCs are in development, mainly for high-risk forms, while in low-risk MDSs, newer approaches target both the regenerative potential of HSCs (i.e., telomerase inhibitors and TPO agonists) or the microenvironment (i.e., TGF-beta signaling inhibitors) [79].

### 3.1. Preclinical Evidence of MSCs’ Role in MDSs

MSCs from MDS patients are functionally altered compared with those from healthy controls (Table 3). In particular, they show reduced expression of cytokines that physiologically express immunomodulatory effects and support hematopoiesis [80]. Ineffective hematopoiesis has been related to MDS–MSC impairment in the production of osteopontin, angiopoietin, Jagged1, and stromal-derived factor 1-CXCL-12, all contributing to HSC support in physiological conditions [81,82,83]. In addition, MSCs from MDSs display genetic abnormalities that are different from those present in the myelodysplastic HSCs [84]. Chromosomal analysis of MSCs revealed karyotype abnormalities in a fraction of MDS/AML patients, but not in healthy controls [85]. These cytogenetic lesions of MSCs are associated with the 5q- syndrome [86] or with the high-risk karyotype of HSCs. However, their pathogenic relevance is still to be addressed and related to MDS-specific altered signaling pathways (e.g., methylation, spliceosoma, proliferation).

#### 3.1.1. MSCs’ Role in Inducing Clonal Hematopoiesis in MDSs

A first indirect observation of the contribution of MSCs to neoplastic evolution comes from the intriguing phenomenon of “donor cell-derived hematopoietic neoplasm”, that is the leukemic transformation of healthy donor HSCs when transplanted in the “sick” BM microenvironment of the leukemic recipient [87]. Raaijmakers et al. demonstrated that mice with deletion of *Dicer1* in osteoprogenitors, but not in hematopoietic cells, developed MDS or AML, whose neoplastic clone did not harbor *Dicer1* deletion [88]. Moreover, *Dicer1* expression has been found to be lower in MSCs from MDS/AML patients compared to healthy controls, impairing the physiologic osteogenic differentiation [89]. In more recent years, the WNT/APC/beta-catenin pathway has been found to be hyperactivated in osteoblasts from MDS/AML patients, and in mouse models, this leads to the leukemic evolution of HSCs via NOTCH pathway activation [90]. As formal genetic proof, transplantation of normal HSCs into irradiated *Apc*-haploinsufficient mice resulted in the development of an MDS-like phenotype [91]. In addition, exposure to pyrvinium, an antihelmintic drug that is also able to block WNT signaling, can inhibit MDS development in *Apc*-deleted mice and patients with 5q- syndrome [92]. Altogether, these experimental models suggest a fundamental role of MDS–MSCs to the emergence of the neoplastic clone (Table 3).

#### 3.1.2. MSCs’ Role in Facilitating Clonal Hematopoiesis in MDSs

MSCs promote MDS development through the creation of an inflammatory milieu. Hyperactivation of inflammatory pathways in MSCs, including NF-κB, EGF, TGF-β, and TNF-α, inhibits hematopoiesis in low-risk MDSs, accounting for the increased apoptotic rate at this stage of the disease [93,94]. Moreover, an inflamed BM niche may favor the expansion of mutated small clones (e.g., *TET2*-mutated ones), such as those accounting for the so-called clonal hematopoiesis of indeterminate potential (CHIP) that would unlikely autonomously evolve to overt myeloid disease [95]. Additionally, MSCs can contribute to the immunity imbalance largely described in MDSs [96]. The latter include the impaired function of NK cells, CD4+ and CD8+ T-cells, and increased activity of Th17. The Treg compartment is expanded in later stages of MDSs [97], possibly accounting for reduced antileukemic immunity, and correlates with higher BM blast infiltration, higher IPSS scores, and disease progression [98]. MSCs may further favor immune escape through the secretion of the immunosuppressive enzyme indoleamine 2,3-dioxygenase (IDO) [99] and via the downregulation of costimulatory molecules CD40, CD80, and CD86 [100]. In MDSs and AML secondary to Fanconi anemia, MSCs exert an immunosuppressive action via increased production of prostaglandins, which are able to reduce T-cell immunity against leukemic cells [101]. More recently, MDS–MSCs were shown to inhibit NK cells, which are pivotal for cancer immune surveillance [102]. Finally, neoplastic MSCs are able to switch from MSC Type-1 (proinflammatory) to “tumor-educated” MSC Type-2 cells (anti-inflammatory) that exhibit stronger immunosuppressive and migratory properties and promote proliferation and drug resistance [103]. On the other hand, neoplastic HSCs may reprogram the microenvironment to favor immune escape. As a matter of fact, healthy MSCs adopt MDS–MSC molecular features when exposed to MDS–HSCs [104]. In addition, some oncogenic mutations commonly seen in the neoplastic HSCs are able to induce a proinflammatory microenvironment, as demonstrated for *TET2*-mutant HSCs [105]. Finally, also aging and toxic insults have been shown to damage MSCs and promote MDS development. When transplanted in an aged microenvironment, HSCs show monoclonality more frequently than those exposed to a young niche [106]. During aging, MSCs display less regenerative ability and a global “senescent” behavior characterized by the secretion of inflammatory cytokines [107]. More recently, the toxicity of iron overload, known to negatively impact on normal hematopoiesis, has been demonstrated to favor mitochondrial fragmentation in MSCs from MDS patients, impairing their functionality [108]. Taken together, these studies highlight that the BM niche has a facilitating rather than initiating effect on neoplastic HSCs (Table 3).

### 3.2. Therapeutic Strategies in MDSs Involving MSCs

There is growing interest in targeting the BM niche, besides the dysplastic HSCs (Table 4). In myeloid malignancies, many attempts are ongoing to inhibit the adhesion of leukemic cells to the stroma by targeting the CXCR4–CXCL12 axis, VLA-4, E-selectin, CD44, and focal adhesion kinase [109,110,111,112,113]. In particular, CXCL12, pivotal in enhancing the homing of CXCR4-expressing HSCs into BM, is produced at higher levels in MDS–MSCs [114], possibly accounting for BM hypercellularity. CXCL12/CXCR4 axis inhibition may be, therefore, of therapeutic relevance in this disease. Another important molecule is CD47, a transmembrane protein belonging to the immunoglobulin superfamily that is involved in a range of processes (apoptosis, proliferation, adhesion, migration, immune response, and angiogenesis). CD47 is ubiquitously expressed in human cells and has been found to be overexpressed in different tumors, where it acts as a “do not eat me” signal, preventing neoplastic cell phagocytosis by macrophages [115]. CD47 is also overexpressed in high-risk MDSs [116], and may thus be a future therapeutic target. On the other hand, preclinical studies demonstrate that most drugs used in MDSs exert an effect on MSCs and on the inflammatory niche. Besides its immunomodulatory effects [96], lenalidomide can regulate chemokines’ and surface molecules’ expression in MSCs, inhibiting HSC migration [117]. Sotatercept, an activin receptor type II ligand trap, modulates the secretion of stromal factors, which inhibit hematopoiesis [118]. Hypometilating agents have shown to regulate adaptive immunity and cytokine secretome in MDSs, both in vivo and in vitro. Azacytidine (AZA) reduces IL-6 production in MDS–MSCs, thus contributing to the recovery of normal hematopoiesis [119] and also through the regulation of extracellular matrix [120]. In addition, the demethylating activity of AZA is also extended to MSCs: AZA treatment induces demethylation and thus increases the expression of SPINT2/HAI-2, which is methylated and silenced in MDSss and AML [121]. Decitabine influences MSC phenotypes, which become able to induce Treg differentiation [122]. On the other hand, other MDS-directed therapies exert a detrimental effect on the BM niche, possibly explaining their adverse effects. For example, the kinase inhibitor rigosertib, investigated in MDSs and in solid tumors, inhibits MSCs and other stromal components of BM, explaining the lack of hematological improvement beyond its antileukemic action [123].

## 4. Conclusions

All the data presented show that MSCs are involved in AA and MDS pathogenesis, either favoring the immune imbalance typical of the diseases, promoting ineffective myelopoiesis, and/or facilitating clonal evolution and leukemogenesis. These actions take place at different levels during the various disease phases: MSCs fail to support hematopoiesis and enhance apoptosis in AA and low-risk/early-stage MDSs, whilst they may boost immune escape of the leukemic clone in high-risk/late-stage of the disease. The impairment of MSCs and, more broadly, of the entire microenvironment is reflected by morphologic, functional, and genetic alterations, as well as by the defects in MSCs’ secretome. In the last decades, omics studies have shown that AA is not only a disease of the immune system and that the HSCs may accumulate genetic lesions and become dysplastic. Conversely, MDSs emerged to be more than a stem cell disease, where (epi)genetic hits lead to disease initiation and progression. The recent insights about MSCs’ role further stress the importance of considering AA/MDS pathogenesis as a multistep complex process, where the niche and the stem cell become evil partners, establishing a proinflammatory mutagenic circle (Figure 1). As a clinical counterpart, the therapies used against the hematopoietic stem cell clones have limited efficacy in MDSs, slowing leukemic evolution rather than eradicating the disease. A deeper knowledge of the microenvironment may, therefore, contribute to therapeutic progress. As a matter of fact, the aplastic/dysplastic niche is highly dynamic, as paradigmatically shown by the shift from type I (proinflammatory) to type II (tumor-educated) MSCs, providing the rationale for the reeducation of MSCs through therapeutic targeting. The latter may be, in fact, only partly restored by healthy donor HSC transplantation, and seems to highly benefit from allogeneic MSC coinfusion. Donor MSCs may, in fact, participate in BM repopulation and, at the same time, exert a deep and long-lasting immunomodulatory effect. Whether the disease resides in the immune microenvironment or in the HSCs, or both, will need further fascinating research and scientific debate.

## Figures and Tables

**Table 1:** Characteristics of mesenchymal stem cells (MSCs) in aplastic anemia (AA).

Reference	N. of Patients	Main Findings
Lu S.H., et al. 2018	-	Deficient or decreased expression of CD106 + MSCs accelerates bone marrow vascularization failure in AA patients
Deng S., et al. 2019	-	The activation of VEGF–Notch pathway can restore the proliferation function of MSCs in AA patients
Li N., et al. 2020	3	The microRNA miR-144-3p is involved in AA pathogenesis. Its targeting may be a therapeutic strategy
Li H., et al. 2017	15	Bone marrow-derived MSCs modulate the levels of T-helper (Th)1, Th2, Th17, and T-regulatory cells, as well as their related cytokines in AA patients
Zhao J., et al. 2019	-	Gingival-derived MSCs markedly improved survival and attenuated histological bone marrow damage in AA murine models
Li J., et al. 2012	-	MSCs from AA patients show differential gene expression profiles associated with bone marrow failure
Huo J., et al. 2020	49	MSCs from AA patients exhibited multifaceted defects in biological characteristics and alterative molecular genetics in the whole genome
Bueno C., et al. 2014	-	MSC cultures from the bone marrow of AA patients display the same phenotype and differentiation potential as their counterparts from normal controls

**Table 2:** Studies on the therapeutic use of mesenchymal stem cells (MSCs) in aplastic anemia (AA), including clinical trials.

Reference	N. of Patients	Main Findings
***Infusion of MSCs in AA patients***		
Xiao Y., et al. 2012	18	Sequential infusions of MSCs may improve hematopoiesis in AA. Six patients responded, 2 reached complete response at 3 months.
Clé D.V., et al. 2015	9	Infusion of allogeneic MSCs in AA is safe but does not improve clinical hematologic response or engraft in recipient bone marrow. Two patients responded at 6 months.
Pang Y., et al. 2017	74	In phase II prospective trial, MSC infusions evoked a response in 28.4% of cases (6.8% complete) in less than one month, with 88% OS at 17 months
***Cotransplantation of MSCs and allogeneic hematopoietic stem cells***		
Xu L-X., et al. 2011 and 2014	5	Haploidentical HSC transplant combined with umbilical cord MSC infusion allows engraftment with 12.5% incidence of severe GVHD and 25% mortality
Li X-H., et al. 2014	17	Combined transplantation of haploidentical HSCs and MSCs on AA without an HLA-identical sibling was safe, reduced the incidence of severe GVHD (23.5%), and lead to good survival (88.2% and 76.5% at 3 and 6 months)
Liu Z., et al. 2017	44	Cotransplantation of MSCs could reduce the risk of graft failure and severe GvHD in haploidentical setting. Acute GVHD occurred in 1/3 of cases and chronic one in 14.6%. The 12-month overall survival was 77.3%
Xu L., et al. 2018	24	MSC and haploidentical HSC cotransplantation is effective and safe, with 50% acute GVHD, not related to gender, age, donor–recipient relations, and patient/donor pair. Patient/donor pair was significantly correlated with chronic GVHD. One year mortality was 20.8%
Zhao M., et al. 2019	25	Peripheral blood HSCs from unrelated and related donor transplants and MSC infusions are effective and safe in AA. No severe acute GVHD and 21.7% chronic GVHD were registered. Overall survival was 84% at 22.9 months (median follow up). The 5-year overall survival rate after transplantation was 83.6% ± 7.5%
Wang H., et al. 2012	6	Cotransplant of haploidentical HSCs and MSCs in children was effective and safe with no severe acute GVHD nor chronic GvHD, and 100% survival at 15 months
Wei W., et al. 2017	25	Cotransplant of haploidentical HSCs and bone marrow-derived MSCs led to engraftment in 100% of cases (median time 12 days, range 11–22 days). Acute GvHD occurred in 64% cases (5 cases grades II–IV), and one patient died of grade IV skin, gut, and liver GvHD. Five cases developed chronic GVHD.
Wang Z., et al. 2019	35	Cotransplant of haploidentical HSCs and MSCs led to hematopoietic reconstitution in 100% of cases (median time 14 days, range 10–22 days). Grade II/IV acute GvHD occurred in 26% cases and 23% developed chronic GVHD. The overall survival rate was 85.71%, with a median of 22 months (range 3.5–37 months).
Yue C., et al. 2018	6	HLA-related donor HSCT and MSC infusion led to sustained, full donor chimerism, with a median time of myeloid/platelet engraftment of 13–15.5 days. One patient died of acute GVHD, and 5 patients were alive after a median follow-up of 21 months (range 17–40.5).
Hinden L., et al. 2019	26	T-cell and NK-cell increased levels may predict a good response to HSCT and MSC cotransplantation. A better response was observed among patients who expressed low levels of IL-6 and IL-22, Th17-related cytokines, prior to therapy.
***Other therapeutic mechanisms for MSCs***		
Liu L.L., et al. 2018	-	Levamisole displayed a significant suppressive effect on the in-vitro adipogenic differentiation of bone marrow-derived MSCs from AA patients
Qu Y., et al. 2018	-	Cyclosporin A suppressed adipogenic differentiation of MSCs by inhibiting interleukin-6 expression in AA

**Table 3:** Pathogenic role of mesenchymal stem cells (MSCs) in myelodysplastic syndromes (MDSs). MDS–MSCs, MSCs from patients with MDS; BM, bone marrow; AML, acute myeloid leukemia.

Reference	N. of Patients	Main Findings
***MSC alterations in MDSs***		
Zhao Z.G., et al. 2012	14	BM-derived MDS–MSCs showed reduced hematopoiesis support function compared to their normal counterparts and impaired capacity to inhibit T-lymphocyte activation and proliferation in vitro.
Ferrer R.A., et al. 2013	-	Cocultures of MDS–MSCs with CD34+ cells from healthy donors resulted in reduced numbers of colony-forming units. Lenalidomide exposure of low-risk MDS–MSCs was able to rescue erythroid and myeloid colony formation.
Geyh S., et al. 2013	106	MDS–MSCs exhibit reduced proliferative capacities and altered expression of key molecules involved in HSC proliferation.
Abbas S., et al. 2019	6	Compared with healthy controls’ MSCs, MDS–MSCs displayed a shift towards increased apoptosis, lower expression of VEGF, SCF, and ANGPT, aberrant expression pattern in the Notch signaling pathway, and increase in Wnt signaling inhibitors.
Azuma K., et al. 2017	5	MDS–MSCs showed some genetic variants with very low allelic frequency (7–8%), such as NF1–G2114D and NF1–G140, not shared by dysplastic HSCs.
Blau O., et al. 2011	43	Cytogenetic aberrations in MSCs were detected in 16% of MDS/AML patients and were different from those observed in the neoplastic HSCs. No chromosomal abnormalities were identified in MSCs of healthy subjects.
Lopez-Villar O., et al. 2009	36	MDS–MSCs display genomic alterations, some of them associated with the 5q- syndrome.
***MSCs induce clonal hematopoiesis in MDSs***		
Raaijmakers M., et al. 2010	-	Deletion of Dicer1, specifically in mouse osteoprogenitors, disrupts hematopoiesis, resulting in MDSs and AML, with neoplastic cells having Dicer1 intact.
Ozdogan H., et al. 2017	10	DICER1 gene expression was lower in MDS–MSCs than healthy controls’ MSCs, and resulted in suppression of the physiologic osteogenic differentiation.
Kode A., et al. 2014	45	An activating mutation of β-catenin in mouse osteoblasts alters the differentiation potential of HSCs via the activation of Notch signaling, leading to the development of AML.
Stoddart A., et al. 2017	-	Loss of 1 copy of Ctnnb1 is sufficient to prevent the development of MDSs in *Apc^del/+^* mice; the alteration of WNT signaling in the BM niche is responsible for the disease.
***MSCs facilitate clonal hematopoiesis in MDSs and immune escape***		
Ping Z., et al. 2019	45	Activation of NF-κB in MDS–MSCs leads to transcriptional overexpression of inflammatory factors, including negative regulators of hematopoiesis.
Chen S., et al. 2016	12	MSCs from low-risk MDS patients display global activation of inflammatory patterns, with increased NF-kB, EGF, TGF-β, and TNF signaling.
Medyouf H., et al. 2014	31	Healthy MSCs acquire MDS–MSC molecular features when exposed to MDS–HSCs and contribute to the propagation of dysplastic HSCs in orthotopic xenografts through the overproduction of N-cadherin, IGFBP2, VEGFA, and LIF.
Zheng Q., et al. 2018	81	Iron overload damages MDS–MSCs via the enhancement of the AMPK/MFF/Drp1 pathway, resulting in increased apoptosis, higher ROS levels, and increased mitochondrial fragmentation compared with MSCs from noniron-overloaded patients.

**Table 4:** Mesenchymal stem cells (MSCs) as a therapeutic target in myelodysplastic syndromes (MDSs).

Reference	N. of Patients	Main Findings
Carter B.Z., et al. 2017	133	Inhibition of focal adhesion kinase decreases MSC-mediated adhesion/migration and viability of MDS/AML cells and prolongs survival in a xenograft murine model.
Wobus M., et al. 2012	-	Lenalidomide modulates expression of cell surface molecules and chemokine secretion of MSCs in vitro, reducing the migration of HSCs.
Iancu-Rubin C., et al. 2013	-	MSCs treated with sotatercept changed their molecular and secretory profile, increasing the expression and secretion of erythropoiesis-stimulating factors in vitro.
Boada M., et al. 2020	35	In vitro treatment with AZA leads to a significant reduction in IL-6 production by the MDS–MSCs.
Wenk C., et al. 2018	-	AZA regulates the expression of extracellular matrix molecules and interferon pathway components, exerting a direct effect on MDS–MSCs and favoring healthy over malignant HSC expansion in vitro.
Roversi F.M., et al. 2019	10	AZA upregulates SPINT2/HAI-2 expression, which is downregulated in MDSs due to methylation in AML/MDS–MSCs in an in-vitro study.
Pang Y., et al. 2019	28	Treatment with decitabine increases the number of MSCs in G2/M phase and powers the ability of MDS–MSCs to induce the differentiation of T-cells into regulatory T-cells in vitro.
Balaian E., et al. 2019	-	Rigosertib exerts inhibitory effects on the stromal components of the osteohematopoietic niche, including MSCs, in a murine model. This may explain the dissociation between antileukemic activity and the absence of hematological improvement.
